# GOMoDo: A GPCRs Online Modeling and Docking Webserver

**DOI:** 10.1371/journal.pone.0074092

**Published:** 2013-09-06

**Authors:** Massimo Sandal, Tran Phuoc Duy, Matteo Cona, Hoang Zung, Paolo Carloni, Francesco Musiani, Alejandro Giorgetti

**Affiliations:** 1 Department of Biotechnology, University of Verona, Ca’ Vignal 1, Verona, Italy; 2 Laboratory of Computational Physics, University of Technology, Vietnam National University, Ho Chi Minh City, Vietnam; 3 Laboratory of Computational Physics, University of Science, Vietnam National University, Ho Chi Minh City, Vietnam; 4 Computational Biophysics, German Research School for Simulation Sciences, Jülich, Germany; 5 Institute for Advanced Simulation, Forschungszentrum Jülich, Jülich, Germany; 6 Scuola Internazionale Superiore di Studi Avanzati (Sissa/ISAS), Trieste, Italy; University of Bologna & Italian Institute of Technology, Italy

## Abstract

G-protein coupled receptors (GPCRs) are a superfamily of cell signaling membrane proteins that include >750 members in the human genome alone. They are the largest family of drug targets. The vast diversity and relevance of GPCRs contrasts with the paucity of structures available: only 21 unique GPCR structures have been experimentally determined as of the beginning of 2013. User-friendly modeling and small molecule docking tools are thus in great demand. While both GPCR structural predictions and docking servers exist separately, with GOMoDo (GPCR Online M odeling and D ocking), we provide a web server to seamlessly model GPCR structures and dock ligands to the models in a single consistent pipeline. GOMoDo can automatically perform template choice, homology modeling and either blind or information-driven docking by combining together proven, state of the art bioinformatic tools. The web server gives the user the possibility of guiding the whole procedure. The GOMoDo server is freely accessible at http://molsim.sci.univr.it/gomodo.

## Introduction

GPCRs are a vast superfamily of eukaryotic transmembrane receptors which act as ubiquitously expressed key regulatory elements and constitute more than 30% of current drug targets [1]. Solving GPCR structures is notoriously technically daunting: as of June 2013, structures for only 21 currently unique GPCRs were available, less than 3% of the GPCR diversity of the human genome. Thus, computational tools are needed to obtain structural information for most GPCR-targeting drug design and/or biophysical studies of receptor/ligand interactions.

All GPCRs share a 7-helix membrane spanning architecture; however the average sequence identity between members of the superfamily is often below 20% [2], making target selection and alignment required for homology modeling far from trivial. Nonetheless, homology modeling has succeeded in predicting ligand-target interactions information for several different GPCRs [3–6]. In many cases, bioinformatic tools aided by experimental validation have been crucial for accurate structural characterization [7–11].

Both GPCR modeling servers/databases (GPCR-SSFE [12], GPCR-ITASSER [13], and GPCR-ModSim [14]) and small ligand docking servers exist (MEDock [15], PatchDock [16], and SwissDock [17]). However, there is a lack of tools that allow users to go seamlessly from sequence to docking, as well as letting users guide the procedure with experimental data. An additional hindrance is that a robust *in silico* approach to drug design and ligand-receptor investigation requires mastering a wide array of software tools, from alignment to modeling to docking and structural refinement. Groups interested in ligand-GPCR structural information do not necessarily have this breadth of structural bioinformatics expertise; furthermore, even users familiar with modeling and docking software may find a quicker and reproducible method very useful. GOMoDo is meant to allow both expert and non-expert users to obtain readily, with minimum effort, biologically and pharmacologically relevant results. GOMoDo, by itself, does not use any novel or untested method, but simply puts together state-of-the-art, proven bioinformatic tools in an easy user interface. The procedure herein automated has been already successfully tested multiple times [8,11,44].

## The GOMoDo Pipeline

**Figure 1:**
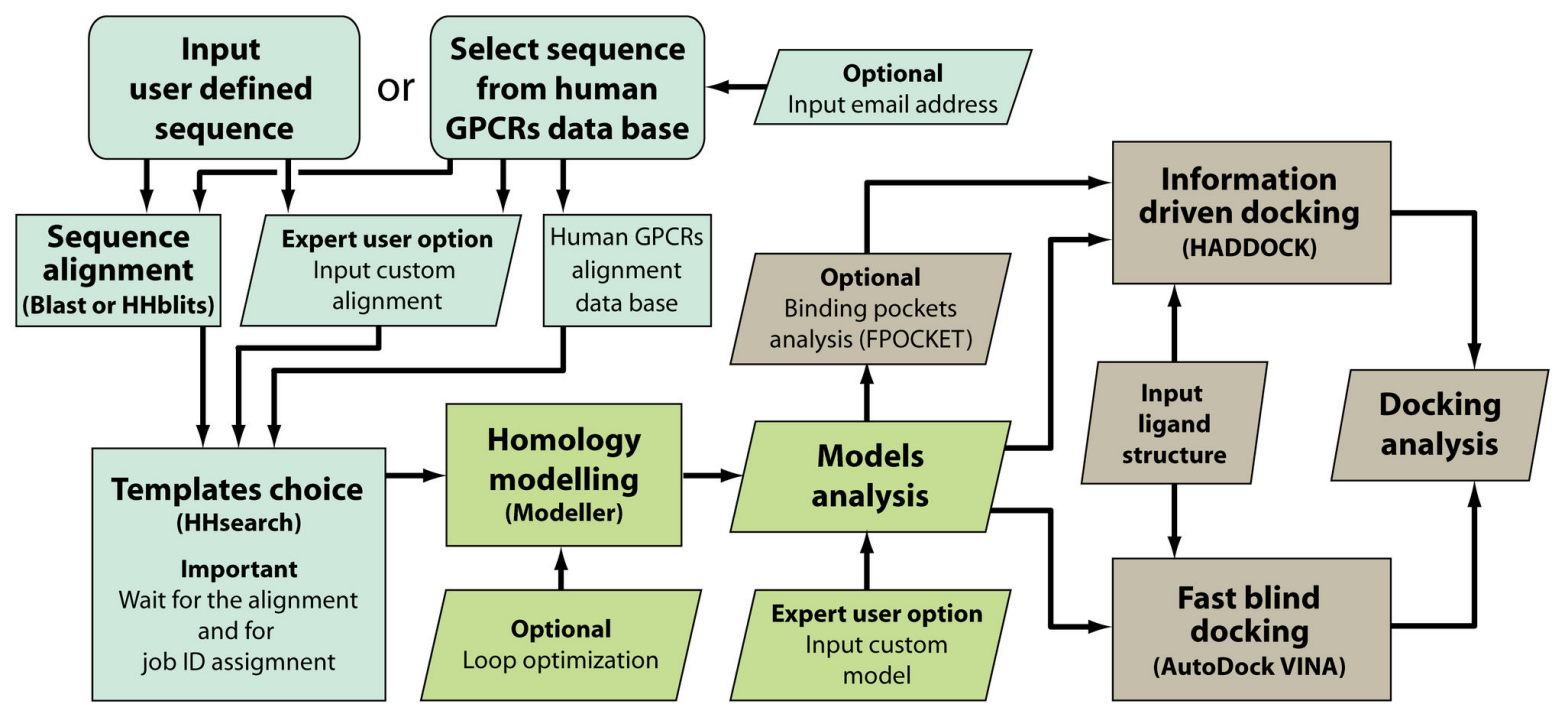
GOMoDo flow chart colored accordingly to the pipeline section. Alignment and template choice, homology modeling and model assessment, and docking sections are reported as light blue, light green and gray, respectively.

The GOMoDo pipeline is briefly resumed in Figure 1. All the programs used in the pipeline make use of the standard parameters, unless noted.

### Alignment and template choice

The server follows two possible routes (Fig. 1). Users can upload a target sequence or can select one of the human GPCRs available in a local database by using the name of the receptor. A standard HHsearch protocol [18,19] is then used to find structurally related templates from remotely homologous sequences. Given that in some cases heuristic algorithms are not accurate enough to obtain a reliable HMM for the target [20], a good alignment is recommended for the generation of a robust HMM profile. For the generation of the HMM, the users can (i) choose to input an alignment of the target with relevant sequences from the same subfamily, (ii) let the server calculate such alignment, or (iii) in the case of human GPCRs, use one of the pre-generated alignment present in the server. For the latter case, sequences of the different GPCRs subclasses, as obtained in the work by Almén and collaborators [21], were aligned by us. We have used the program PROMALS [22], following the same methodology as in refs. 8,11. If a user- or database-provided alignment is not available, the initial multiple sequence alignment is automatically generated by a search of similar sequences, using either classic BLAST [23] or the HMM-based search with HHblits [24]. The user can choose the number of rounds of sequence search in either case. Secondary structure information is added to the alignment using PSI-PRED [25]. A hidden Markov model (HMM) is then built and calibrated from the multiple alignment using HHmake [19], matched to suitable templates of known structure with HHsearch [19] using the database of HMMs available at ftp://toolkit.lmb.uni-muenchen.de/pub/HHsearch/databases/. Suitable templates are chosen from a complete set of GPCRs three-dimensional structures (see Table S1 for a list of PDB codes of available templates as of manuscript submission). Templates and the corresponding HMM database are updated every 1-2 months. Finally a structural alignment is produced for each template using HHmakemodel. Before proceeding to modeling, we allow the users to check the correctness of the alignment with JALVIEW [26]. This procedure has been shown previously to obtain template/target alignments useful for homology modeling whenever the sequence similarity is low but the overall fold similarity is high, as in the case of GPCRs [8,11].

### Homology modeling and model quality assessment

A user-chosen number of models is made for each template using MODELLER9v10 [27] with standard single-template parameters (Figure 1, users must have their own MODELLER key). The user can also choose for automatic loop refinements to be performed for each model by means of the standard *loopmodel* class. For each model, GOMoDo outputs the template PDB code, DOPE score (full and normalized) [28], MOLPDF score [27] and GA341 score [29] as well as other information A scatter plot of the GA341 *vs.* normalized DOPE score for all models is available. Furthermore, models can be directly submitted to the VADAR model quality assessment server [30]. By using VADAR users can check more than 30 model quality indicators, which include structural descriptors calculated by DSSP [31], WHATIF [32] or PROCHECK [33] like the Ramachandran plot, fractional accessible surface area, fractional volume, 3D quality index and stereo/packing quality index. Upon completion of the modeling job, the user is notified by an automatic e-mail sent by the server. Alternatively, the modeling results can be retrieved directly from the webserver up to 60 days later, by inserting the modeling job ID and/or e-mail address. Alignment and modeling together can take from several tens of minutes to several hours, depending on server load and the number of requested homology models.

Advanced users may prefer to refine or modify the GOMoDo-obtained models by themselves before proceeding to docking. We allow users to upload custom models along with the ones obtained in the output, and to compare them before proceeding. In order to facilitate the user, we include a page with the web links of other GPCRs modeling online tools.

### Docking

GOMoDO can dock small molecules and peptides (i) blindly (using AutoDock VINA [34], herein referred as VINA) or (ii) using experimental information (using HADDOCK [35,36]) as reported in Figure 1.

(i) VINA [34] is a fast, multithreaded and accurate rewriting of AutoDock that often outperforms classical AutoDock both in speed and quality [37,38]. A three-dimensional SDF or PDB file of the ligand is all the input required. We also provide a library of olfaction-related compounds from the OlfactionDB database [39]. On the GOMoDo server, we can return ten VINA-docked structures in less than a minute. VINA requires knowledge of the location search space for conformations of the ligand --- in other words, the generic location of the binding site. The server already contains such information for all templates with respect to the template PDB coordinate system; therefore before docking, the model is structurally aligned with the templates using LOVOALIGN [40] to guarantee that the search space box is in the correct position with respect to the model coordinate system. In simple cases (small ligands and targets close in sequence to a modeling template) GOMoDo with VINA can yield quick and reasonable results.
(ii) Available experimental information on the residues involved in ligand binding such as NMR titration experiments or mutagenesis data can be used to guide docking. This can be of crucial importance when docking is based on non-trivial homology models, as is often the case for GPCRs. We therefore offer an interface to the HADDOCK software [35,36], where the user can indicate explicitly the protein residues involved in the receptor-ligand interaction. HADDOCK performs a slower but more refined docking than VINA. In particular, HADDOCK includes a final refinement step with molecular dynamics in explicit water which allows for flexibility of specified residues. This feature has the further positive side effect of including side-chain optimization of the binding cavity. While advanced users can take advantage of the online official HADDOCK server [41] for this (which gives full control over all docking options), we also offer a simplified in-house interface to the HADDOCK software that allows the docking of ligands, peptides or interacting proteins. If the ligand is a small non-protein molecule, HADDOCK requires parameter files with partial charges for the ligand, as well as CNS parameter files. Users can obtain them from PRODRG [42] or produce them using their own calculations. GOMoDo allows downloading the entire HADDOCK output either as a compressed archive, individual clusters or single output structures. HADDOCK advanced refinement is also attractive when experimental information is missing. To use HADDOCK for blind docking in GOMoDo, the user can analyze the model on the server with FPOCKET [43] and obtain in a few seconds predictions of plausible binding pockets; in the Supplementary Information, we give two successful examples of this usage (Figure S1). FPOCKET is also useful for obtaining accessory information for experiment-guided docking.

## Application Cases

The methodology automatized in GOMoDo has been already used to structurally characterize ligand-GPCRs adducts [8,11,44]. To assess the server, we tested GOMoDo by reproducing selected examples of known GPCR-ligand complexes present in the PDB (targets). In every case we mimicked what a standard user would do, as advised by the manual. For the selected PDB complexes we took the full protein sequence from UniProt and generated 30 models per template using the default BLAST alignment for HMM generation. We then picked up the best model according to DOPE and GA341 scores, obviously excluding models built on the structure of the target. The ligands present in the crystal structure were then docked to the model using VINA or HADDOCK programs.

**Figure 2:**
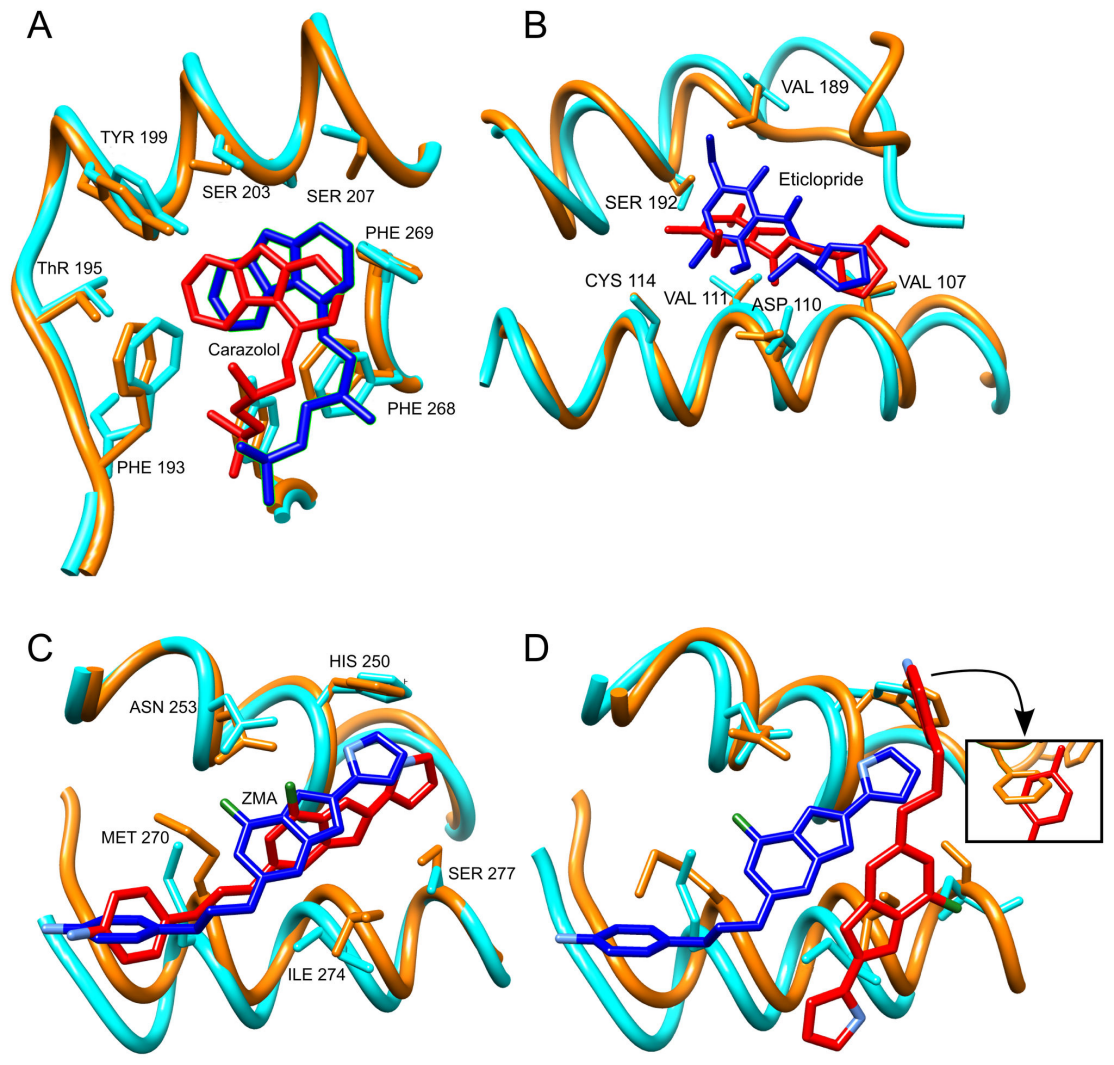
Reproducing GPCR-ligand complexes with GOMoDo. hβ2AR (**A**), hD3R (**B**) and hA _2_AR (**C**,**D**) binding sites in the immediate neighbourhood of ligands for crystal structures and models obtained with GOMoDo. The homology model and the docked ligand are orange and red, respectively. The experimental structure and ligand are cyan and blue, respectively. (**A**) VINA docking of carazolol to the hβ2AR model and compared to the crystal structure (PDB: 2RH1). (**B**) VINA docking of eticlopride to the hD3R model and compared to the crystal structure (PDB: 3PBL). HADDOCK (**C**) and VINA (**D**) docking of ZMA to the hA _2_AR compared with the crystal structure (PDB: 3EML). Insert in **D** shows the unphysical artefacts generated by the VINA docking.

Here we describe three examples of applications (Figure 2): the first example is a simple application using the human beta-2 adrenergic receptor (hβ2AR) that has been previously used by some of us as a test case [44]. The second example is the modeling of human dopamine D3 receptor (hD3R), a slightly more complex case because of the low sequence identity between the receptor and the templates. Finally, the third example deals with the human A_2_A adenosine receptor (hA _2_AR) in complex with the antagonist ligand ZM241385 (ZMA). In this case, some difficulties can be expected because the antagonist is a rather bulky molecule. Other application examples, which can be found in Figure S1, deal with the human histamine H1 receptor and human kappa-opioid receptor.

### hβ2AR

The structure of the hβ2AR (UniProt ID: P07550) was modeled using as a template the structure of the turkey beta-1 adrenergic (tβ1AR) receptor (PDB entry: 2Y00 [45], UniProt ID: P07700). The sequence identity between the two receptors is about 46.1% and thus the model is indeed of relatively good quality [Cα root mean square deviation (rmsd) = 1.3 Å]. Furthermore, the ligand is relatively small and thus it probably does not require significant conformational changes of the side chains to fit in the binding cavity. We docked the ligand carazolol to the hβ2AR model with fast VINA and compared our results against the experimentally determined X-ray structure (PDB code: 2RH1 [46]). The resulting structure of the ligand-receptor complex is in fair agreement with the crystal structure, in terms of both the ligand pose and the side-chains orientation (Figure 2A, rmsd calculated on heavy atoms = 2.9 Å).

### hD3R

VINA can also give reasonable results when used with distant homology models. We modelled hD3R (UniProt ID: P35462) in complex with the antagonist eticlopride, and compared the results with the corresponding crystal structure (PDB code: 3PBL [47]). The best template in this case was human sphingosine-1 phosphate receptor (PDB code: 3V2Y [48], UniProt ID: P21453), with a sequence identity of 22.1%. Despite the relatively poor modelling of loops in the binding site, the orientation of the binding pose is correct (Figure 2B, rmsd = 4.0 Å).

### hA_2_AR

An example of a harder task is reproducing the structure of hA _2_AR in complex with ZMA, whose crystal structure is available as PDB code 3EML [49] (UniProt ID: P29274). Here, the best template, which happens to be again the tβ1AR (PDB: 2Y00) structure, shares only 28.1% of sequence identity with the target. Hence, the model can be expected to be less accurate (model/target Cα rmsd = 4.0 Å) than the one previously described. In this case, the antagonist is also significantly bulkier. We therefore used HADDOCK [35,36] and we set inter-molecular interactions between the ligand and residues Asn253, Met270, Ile274, Ser277, and His250 as active restraints. We also used the best FPOCKET predicted binding pocket - which encompassed the binding residues - as passive restraints (that is, the residues that HADDOCK is given permission to move to accommodate the ligand). Default options of HADDOCK and default PRODRG settings for ligand were used. The best structure of the most populated HADDOCK-derived cluster, (Figure 2C) is very similar to that of the X-ray structure (rmsd = 2.1 Å) [49].

The third case (the modeling of hA _2_AR) shows that advanced docking methods are often required to obtain reasonable structures. Docking of ZMA to the hA _2_AR model structure with VINA fails: the ligand is shifted from the correct binding site and flipped by 180 degrees on two axes (Figure 2D, rmsd = 10.5 Å). Moreover, even the VINA best structure in this case shows gross clashes between the ligand and protein side chains due to the difficulty in accommodating the ligand while treating the protein as rigid (Figure 2D, insert). Notice that even if the starting model is the same for VINA and HADDOCK, the backbone and side chain orientation of models is slightly different and often closer to the experimental structure in the latter. This is due to the flexible refinement of HADDOCK performed in explicit solvent, while VINA treats the model as rigid. In Figures S1A and S1B, we show how HADDOCK can also be useful in the absence of experimental information by using FPOCKET to obtain restrains.

## Conclusions

GOMoDo is intended to be a user friendly pipeline that puts together several state-of-the-art tools and allows experienced and inexperienced users to obtain GPCR’s homology models together with predictions of ligand binding poses. GOMoDo was developed to be flexible and adaptable to the user’s demands. For this reason, every step of the GOMoDo pipeline allows user intervention (if needed) to i) insert alignments, ii) use homology models generated by other methods, iii) predict binding cavities and iv) include experimental restraints for performing knowledge-based virtual docking experiments. The combination of all these tools in a single publicly available web server is GOMoDo’s novelty. In particular, the possibility of interacting with the server all along the pipeline allows the user to include experimental information from molecular biology experiments into the process, preventing it from becoming a fully-automatic black-box.

## Supporting Information

Table S1 **GPCR structures available as templates in GOMoDo as of June 2013.** Note that the template database is regularly updated every 1-2 months.
(DOCX)

Figure S1 **Further examples of GOMODO in action.** Receptor binding sites in the immediate neighbourhood of ligands for crystal structures and models obtained with GOMoDo. The homology model and the docked ligand are orange and red, respectively. The experimental structure and ligand are cyan and blue, respectively.
Here we show examples of successful *blind* HADDOCK docking, exploiting FPOCKET to guess residues involved in the binding cavity. All the residues corresponding to the best FPOCKET-calculated binding cavity were used as both active and passive restraints. (**A**) Human histamine H1 receptor (UniProt ID: P35367) in complex with *trans-*doxepin: model and docking compared with crystal structure (PDB code: 3RZE). Model template is human M2 muscarinic acetylcholine receptor (PDB code: 3UON, UniProd ID: P08172). (**B**) Human kappa-opioid receptor (UniProt ID: P41145) in complex with the bulky and flexible ligand JDTic: model and docking compared with crystal structure (PDB code: 4DJH). Model template is the mouse μ-opioid receptor (PDB structure: 4DKL, UniProt ID: P42866). In this case the pose is slightly shifted with respect to the crystal structure and rotameric state is different; however position and global orientation are correct. Here nitrogen atoms are in dark green and oxygen atoms in cornflower blue.
(TIF)
