# Supplementary material for: GOMoDo: A GPCRs Online Modeling and Docking Webserver
Source: PLoS One. 2013 Sep 6;8(9):e74092. doi: 10.1371/journal.pone.0074092 (PMC3772745; doi:10.1371/journal.pone.0074092)
Supplement: Table S1 — GPCR structures available as templates in GOMoDo as of June 2013. Note that the template database is regularly updated every 1-2 months. (DOCX) [file pone.0074092.s001.docx]

**GOMoDo: a GPCRs online modeling and docking webserver**

Massimo Sandal, Tran Phuoc Duy, Matteo Cona, Hoang Zung, Paolo Carloni,
Francesco Musiani and Alejandro Giorgetti

**SUPPLEMENTARY TABLE S1**

**Supplementary Material**

**Table S1.** GPCR structures available as templates in GOMoDo as of June 2013 (part 1).

| **PDB**  **code** | **Protein name** | **Ligand** | **State** | **Species** | **Resolution**  **(Å)** |
| --- | --- | --- | --- | --- | --- |
| 1F88 | Rhodopsin | 11-cis retinal | inactive | *B. taurus* | 2.80 |
| 1L9H | Rhodopsin | 11-cis retinal | inactive | *B. taurus* | 2.60 |
| 1GZM | Rhodopsin | 11-cis retinal | inactive | *B. taurus* | 2.65 |
| 1U19 | Rhodopsin | 11-cis retinal | inactive | *B. taurus* | 2.20 |
| 2J4Y | Rhodopsin | 11-cis retinal | inactive | *B. taurus* | 3.40 |
| 2I37 | Rhodopsin | None | photoactivated | *B. taurus* | 4.15 |
| 3CAP | Rhodopsin | None | opsin | *B. taurus* | 2.90 |
| 3PXO | Rhodopsin | all-trans retinal | Meta II state | *B. taurus* | 3.00 |
| 3PQR | Rhodopsin | all-trans retinal | Meta II state in complex  with C-terminal fragment of GαCT2 | *B. taurus* | 2.85 |
| 3DQB | Rhodopsin | *None* | Ops*-GαCT peptide complex | *B. taurus* | 3.20 |
| 4A4M | Rhodopsin | all-trans retinal | constitutively active meta-II state | *B. taurus* | 3.30 |
| 2Z73 | Rhodopsin | 11-cis retinal | inactive | *T. pacificus* | 2.50 |
| 2ZIY | Rhodopsin | 11-cis retinal | inactive | *T. pacificus* | 3.70 |
| 3AYN | Rhodopsin | 9-cis retinal | Isorhodopsin (artificial) | *T. pacificus* | 2.70 |
| 2VT4 | β-1 adrenergic receptor | cyanopindolol | with antagonist | *M. gallopavo* | 2.70 |
| 2Y00 | β-1 adrenergic receptor | dobutamine | with partial agonist | *M. gallopavo* | 2.50 |
| 2YCW | β-1 adrenergic receptor | carazolol (t1118) | with antagonist | *M. gallopavo* | 3.00 |
| 2YCX | β-1 adrenergic receptor | cyanopindolol (t148) | with antagonist | *M. gallopavo* | 3.25 |
| 2YCZ | β-1 adrenergic receptor | lodocyanopindolol (t756) | with antagonist | *M. gallopavo* | 3.65 |
| 4AMJ | β-1 adrenergic receptor | carvedilol | with biased agonist | *M. gallopavo* | 2.30 |
| 4AMI | β-1 adrenergic receptor | bucindolol | with biased agonist | *M. gallopavo* | 3.20 |
| 2R4R | β-2 adrenergic receptor | carazolol | with inverse-agonist | *H. sapiens* | 3.40 |
| 3KJ6 | β-2 adrenergic receptor | carazolol | with inverse-agonist | *H. sapiens* | 3.40 |
| 2RH1 | β-2 adrenergic receptor | carazolol | with inverse-agonist | *H. sapiens* | 2.40 |
| 3D4S | β-2 adrenergic receptor | timolol | with partial inverse-agonist | *H. sapiens* | 2.80 |
| 3P0G | β-2 adrenergic receptor | BI-167107 | nanobody-stabilized active state | *H. sapiens* | 3.50 |
| 3PDS | β-2 adrenergic receptor | FAUC50 | with irreversibly-bound agonist | *H. sapiens* | 3.50 |
| 3SN6 | β-2 adrenergic receptor | BI-167107 | active state in complex with Gs | *H. sapiens* | 3.20 |
| 3EML | A2A adenosine receptor | ZM241385 | with antagonist | *H. sapiens* | 2.60 |
| 3QAK | A2A adenosine receptor | UK-432097 | with agonist | *H. sapiens* | 2.71 |
| 2YDO | A2A adenosine receptor | adenosine | with agonist | *H. sapiens* | 3.00 |
| 2YDV | A2A adenosine receptor | NECA | with agonist | *H. sapiens* | 2.60 |
| 3RFM | A2A adenosine receptor | caffeine | with inverse-agonist | *H. sapiens* | 3.60 |
| 4EIY | A2A adenosine receptor | ZM241385 | with antagonist | *H. sapiens* | 1.80 |
| 3VG9 | A2A adenosine receptor | ZM241385 | in complex with inverse-agonist antibody | *H. sapiens* | 2.70 |
| 2LNL | CXCR1 chemokine receptor | *None* | Physiological in-membrane state | *H. sapiens* | NMR |
| 3ODU | CXCR4 chemokine receptor | IT1t | with antagonist | *H. sapiens* | 2.50 |
| 3PBL | Dopamine D3 receptor | eticlopride | with antagonist | *H. sapiens* | 2.89 |
| 3RZE | Histamine H1 receptor | doxepin | with antagonist | *H. sapiens* | 3.10 |

(continue)

**Table S1.** GPCR structures available as templates in GOMoDo as of June 2013 (part 2).

| **PDB**  **code** | **Protein name** | **Ligand** | **State** | **Species** | **Resolution**  **(Å)** |
| --- | --- | --- | --- | --- | --- |
| 3V2W | Sphingosine 1-phosphate receptor | ML056 | with antagonist | *H. sapiens* | 3.35 |
| 3V2Y | Sphingosine 1-phosphate receptor | ML056 | with antagonist | *H. sapiens* | 2.80 |
| 3VW7 | Protease activated receptor 1 | Vorapaxar | with antagonist | *H. sapiens* | 2.20 |
| 3UON | M2 muscarinic acetylcholine receptor | 3-quinuclidinyl-benzilate | with antagonist | *H. sapiens* | 3.00 |
| 4DAJ | M3 muscarinic acetylcholine receptor | Tiotropium | with antagonist | *H. sapiens* | 2.90 |
| 4DJH | Kappa-opioid receptor | JDTic | with antagonist | *H. sapiens* | 2.90 |
| 4DKL | Mu-opioid receptor | beta-funaltrexamine | with antagonist | *M. musculus* | 2.80 |
| 4EJ4 | Delta-opioid receptor | naltrindol | with antagonist | *M. musculus* | 3.40 |
| 4EA3 | Nociceptin/orphanin FQ (N/OFQ) receptor | C-24 (peptide) | with antagonist | *H. sapiens* | 3.01 |
| 4GRV | Neurotensin receptor 1 | Neurotensin (8-13) | with agonist | *R. norvegicus* | 2.80 |
