# Supplementary figures and images for: GOMoDo: A GPCRs Online Modeling and Docking Webserver
Source: PLoS One. 2013 Sep 6;8(9):e74092. doi: 10.1371/journal.pone.0074092 (PMC3772745; doi:10.1371/journal.pone.0074092)

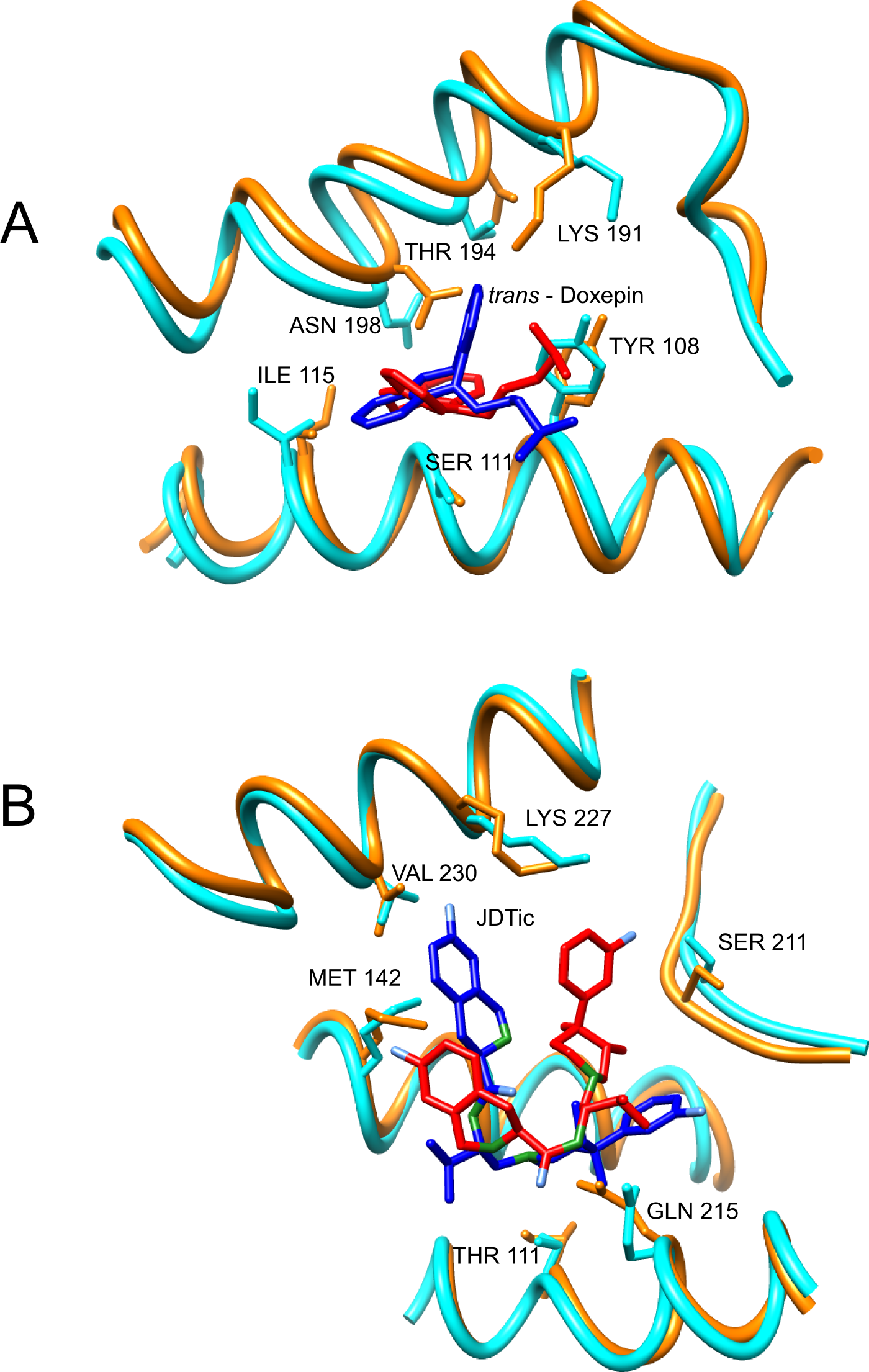

Supplement: Figure S1 — Further examples of GOMODO in action. Receptor binding sites in the immediate neighbourhood of ligands for crystal structures and models obtained with GOMoDo. The homology model and the docked ligand are orange and red, respectively. The experimental structure and ligand are cyan and blue, respectively. Here we show examples of successful blind HADDOCK docking, exploiting FPOCKET to guess residues involved in the binding cavity. All the residues corresponding to the best FPOCKET-calculated binding cavity were used as both active and passive restraints. (A) Human histamine H1 receptor (UniProt ID: P35367) in complex with trans-doxepin: model and docking compared with crystal structure (PDB code: 3RZE). Model template is human M2 muscarinic acetylcholine receptor (PDB code: 3UON, UniProd ID: P08172). (B) Human kappa-opioid receptor (UniProt ID: P41145) in complex with the bulky and flexible ligand JDTic: model and docking compared with crystal structure (PDB code: 4DJH). Model template is the mouse μ-opioid receptor (PDB structure: 4DKL, UniProt ID: P42866). In this case the pose is slightly shifted with respect to the crystal structure and rotameric state is different; however position and global orientation are correct. Here nitrogen atoms are in dark green and oxygen atoms in cornflower blue. (TIF) [file pone.0074092.s002.tif]
